# Supplementary material for: Alpha kinase 1 controls intestinal inflammation by suppressing the IL-12/Th1 axis
Source: Nat Commun. 2018 Sep 18;9:3797. doi: 10.1038/s41467-018-06085-5 (PMC6143560; doi:10.1038/s41467-018-06085-5)
Supplement: Supplementary file 1 — Supplementary Information [file 41467_2018_6085_MOESM1_ESM.pdf]

**Supplementary Information**

Alpha kinase 1 controls intestinal inflammation by suppressing the IL-12/Th1 axis
Ryzhakov et al.

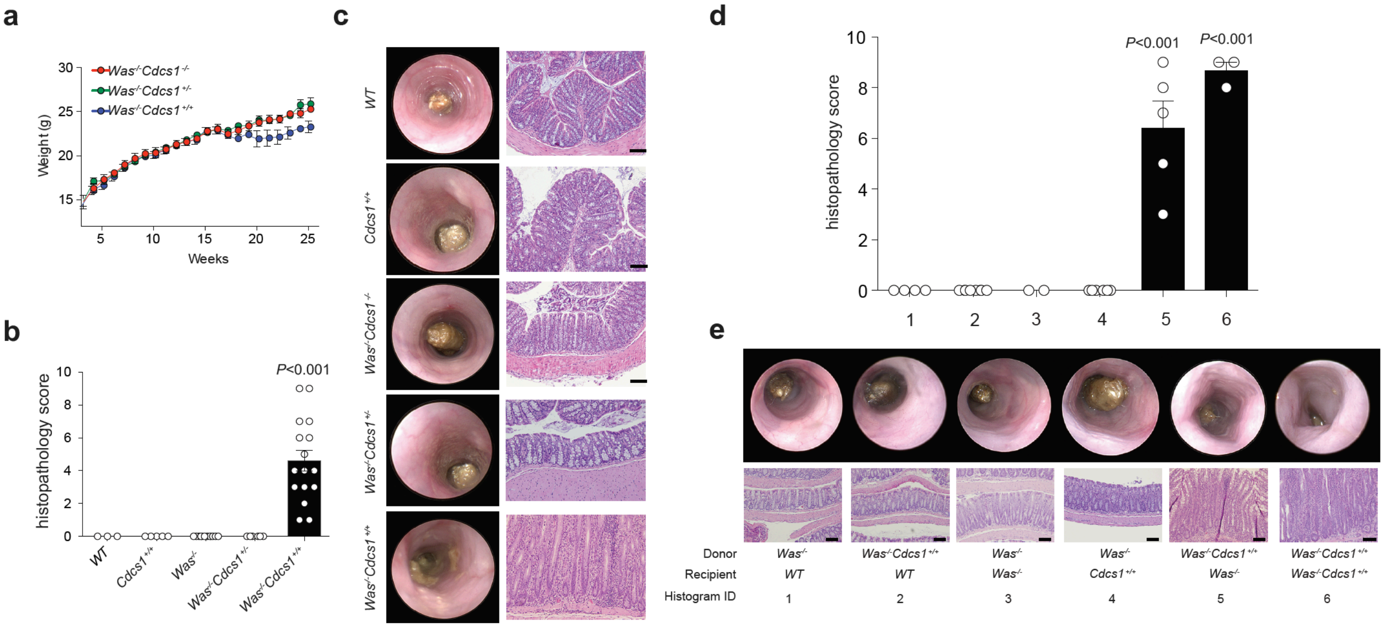


**Supplementary Figure 1. Homozygosity at the *Cdcs1* locus confers colitis susceptibility to *Was*$^{-/-}$ mice on the C57BL/6 background.**

(a) Weight curve for WASP-deficient littermate female mice harboring the congenic C57BL/6 locus (n=5), heterozygous for the *Cdcs1* locus (n=9), or homozygous for the *Cdcs1* locus (n=5).

(b) Combined histological scores on H&E stained formalin-fixed, paraffin-embedded colon tissue. Each circle represents an individual mouse across 3 separate experiments. Error bars represent SEM. Data were analyzed by one-way ANOVA with Dunnett's multiple comparisons test.

(c) Representative endoscopic (left) and histologic (right) findings in WT, *Cdcs1*$^{+/+}$, *Was*$^{-/-}$*Cdcs1*$^{-/-}$, *Was*$^{-/-}$*Cdcs1*$^{+/-}$, and *Was*$^{-/-}$*Cdcs1*$^{+/+}$ mice. Images are 20X with scale bars = 100 µm.

(d-e) *Cdcs1* functions in hematopoietic lineages to confer colitis susceptibility in *Was*$^{-/-}$ mice on the C57BL/6 background. 6-week old recipient mice were irradiated followed by transplantation of donor bone marrow as indicated in panel (e). (d) Colitis score of H&E stained colon sections taken 16 weeks post-transplant with each dot representing an individual animal and combined across 2 independent experiments. Error bars represent the SEM. Data were analyzed by one-way ANOVA with Tukey's multiple comparisons test. (e) Endoscopic evaluation was performed 8 weeks post-transplant (top) with representative colon histological sections shown (bottom). Images are 20X with scale bars = 100 µm.

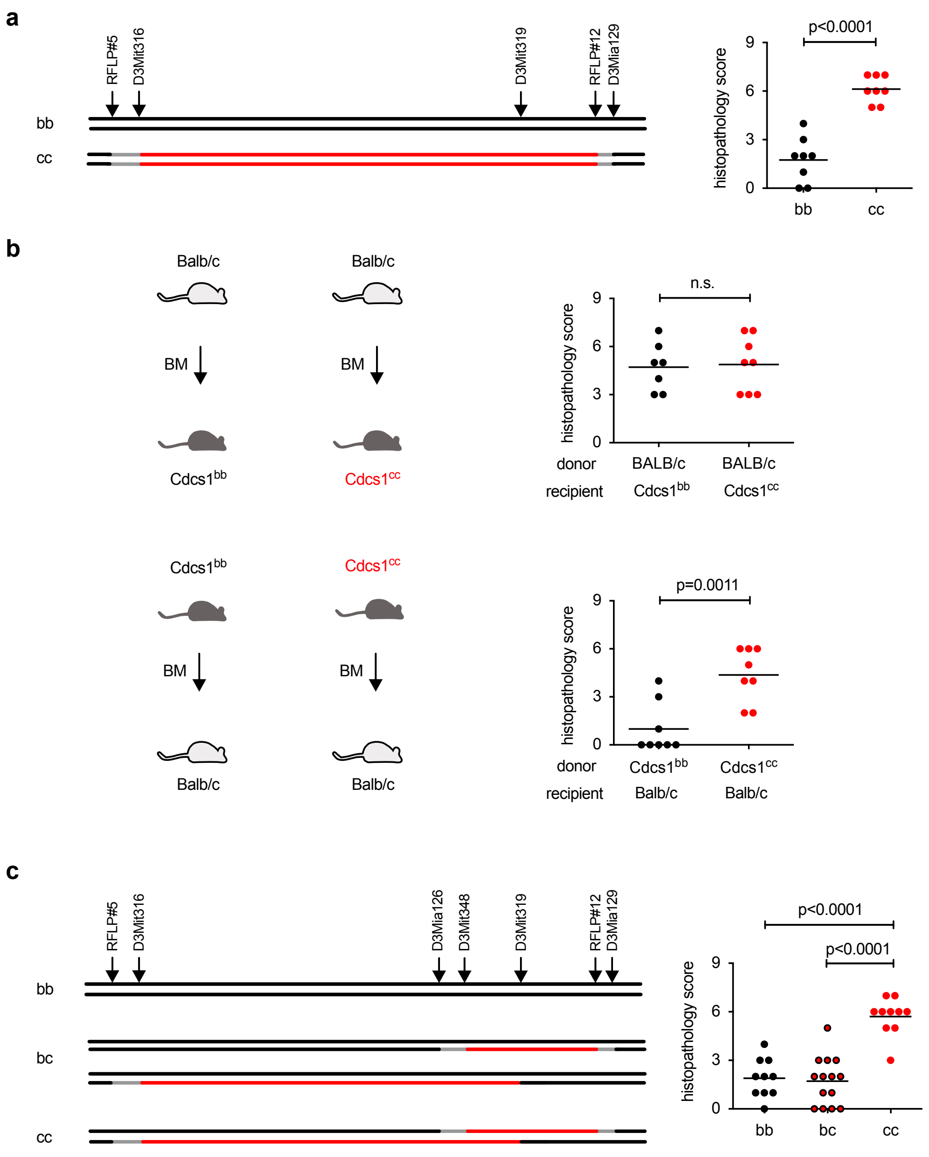


**Supplementary Figure 2. Finemapping of the colitogenic Cdcs1 locus.**

(a-b) The *Cdcs1* locus (D3Mit348-D3Mit319) controls disease severity in TRUC colitis. (a) TRUC mice heterozygous for the extended congenic interval D3Mit316-D3Mit319 from C3H on a C57BL/6 background (*Cdcs1*$^{bc(316\text{-}319)}$) were mated to generate TRUC.*Cdcs1*$^{bb}$ (bb) and TRUC.*Cdcs1*$^{c(316\text{-}319)c(316\text{-}319)}$ (cc) littermates. Mice were sacrificed at 8 weeks of age, and colon histopathology was blinded scored by a GI pathologist. Data are scores of individual mice (n = 8 mice per group), bars represent mean, p value determined by t test. (b) *Cdcs1* exerts its effect on colitis severity through the hematopoietic compartment. (Upper panel) 6 week-old TRUC.*Cdcs1*$^{bb}$ and TRUC.*Cdcs1*$^{c(316\text{-}319)c(316\text{-}319)}$ (Cdcs1$^{cc}$) recipients were lethally irradiated and injected i.v. with BM cells from BALB/c TRUC mice. (Lower panel) Reciprocal experiment using BALB/c TRUC mice as recipients. BM cells were from TRUC.*Cdcs1*$^{bb}$ and TRUC.*Cdcs1*$^{c(316\text{-}319)c(316\text{-}319)}$ respectively. Animals were sacrificed 20 weeks after bone marrow transplantation and colon histology was analyzed (right panels). (c) The interval controlling disease severity in TRUC mice maps to a 1.2–2.1 Mbp chromosomal region coinciding with *Hiccs*. Heterozygous TRUC.*Cdcs1*$^{bc}$ mice with overlapping C3H-derived Cdcs1$^{c}$ intervals were mated to generate Cdcs1$^{bb}$ (bb), Cdcs1$^{bc}$ (bc) , and Cdcs1$^{cc}$ (cc) littermates. Cdcs1$^{cc}$ mice are compound heterozygotes that are homozygous for the overlapping congenic interval as shown. (Lower panel) Animals were sacrificed at 8 weeks of age and colon histopathology was analyzed. Data are scores of individual mice, bars represent mean (n=9–14 mice per group), *p* value determined by t test.

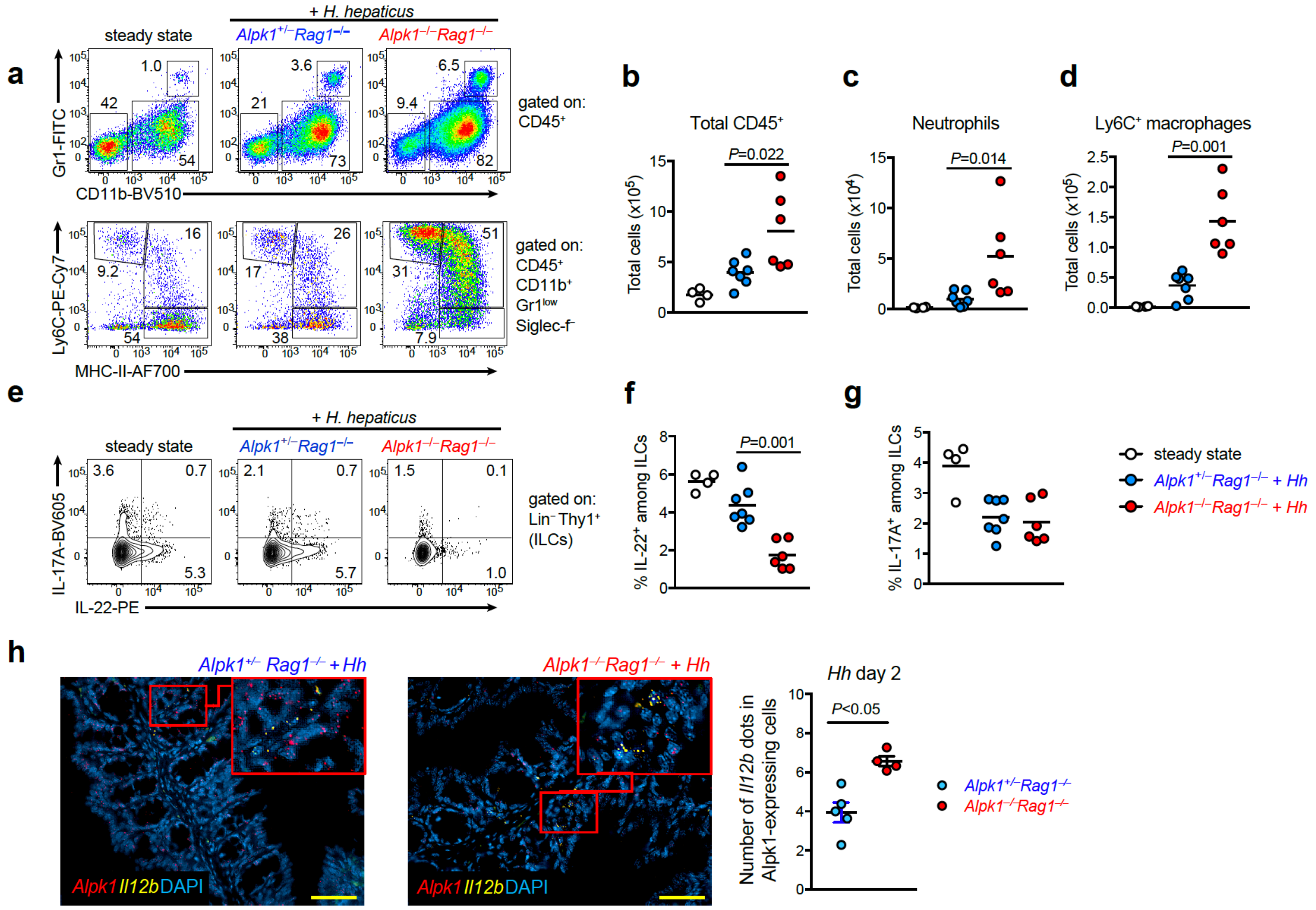

**Supplementary Figure 3. Analysis of immune response in lamina propria during innate *Hh* driven colitis (associated with Fig. 2).**

(a–g) Flow cytometry analysis of colon lamina propria leukocytes (LPL). Data summarize two independent experiments: steady state (n=4), *Hh*-infected B6.*Rag1*$^{-/-}$*Alpk1*$^{-/-}$ (n=6), and *Hh*-infected B6.*Rag1*$^{-/-}$*Alpk1*$^{+/-}$ (n=7). In all panels, statistics represent Mann-Whitney tests for *Hh*-treated groups only.

(a) Representative flow cytometry plots displaying neutrophil (CD11b$^{high}$ Gr1$^{high}$), inflammatory monocyte (Ly6C$^{high}$ MHCII$^{-}$), and inflammatory macrophage (Ly6C$^{high}$ MHCII$^{+}$) frequencies in colon lamina propria leukocytes (LPL) at steady state and after 12 days of *Hh* infection.

(b–d) Total numbers of CD45$^{+}$ LPL, neutrophils, and inflammatory macrophages.

(e) Representative FACS plots displaying IL-17A and IL-22 production by colonic ILCs.

(f–g) Frequency of IL-22 and IL-17A expression among Lin$^{-}$Thy1$^{+}$ lymphocytes.

(h) *Il12b* (yellow) and *Alpk1* (red) mRNA expression *in situ* in colon tissue of B6.*Rag1*$^{-/-}$*Alpk1*$^{-/-}$ (n=4) and B6.*Rag1*$^{-/-}$*Alpk1*$^{+/-}$ (n=5) mice infected with *Hh* for two days. Fluorescent staining of formalin-fixed tissues was carried out using RNAScope® according to the manufacturer's protocol. Nuclear staining (DAPI) is shown in blue. Scale bars (yellow) = 50 µm. The overall quantification of *Il12b* mRNA dots per cell averaged per mouse is shown on the right panel. A minimum of 10 individual cells per mouse and per condition was used for quantification. One experiment was performed, data were analysed by Mann-Whitney test.

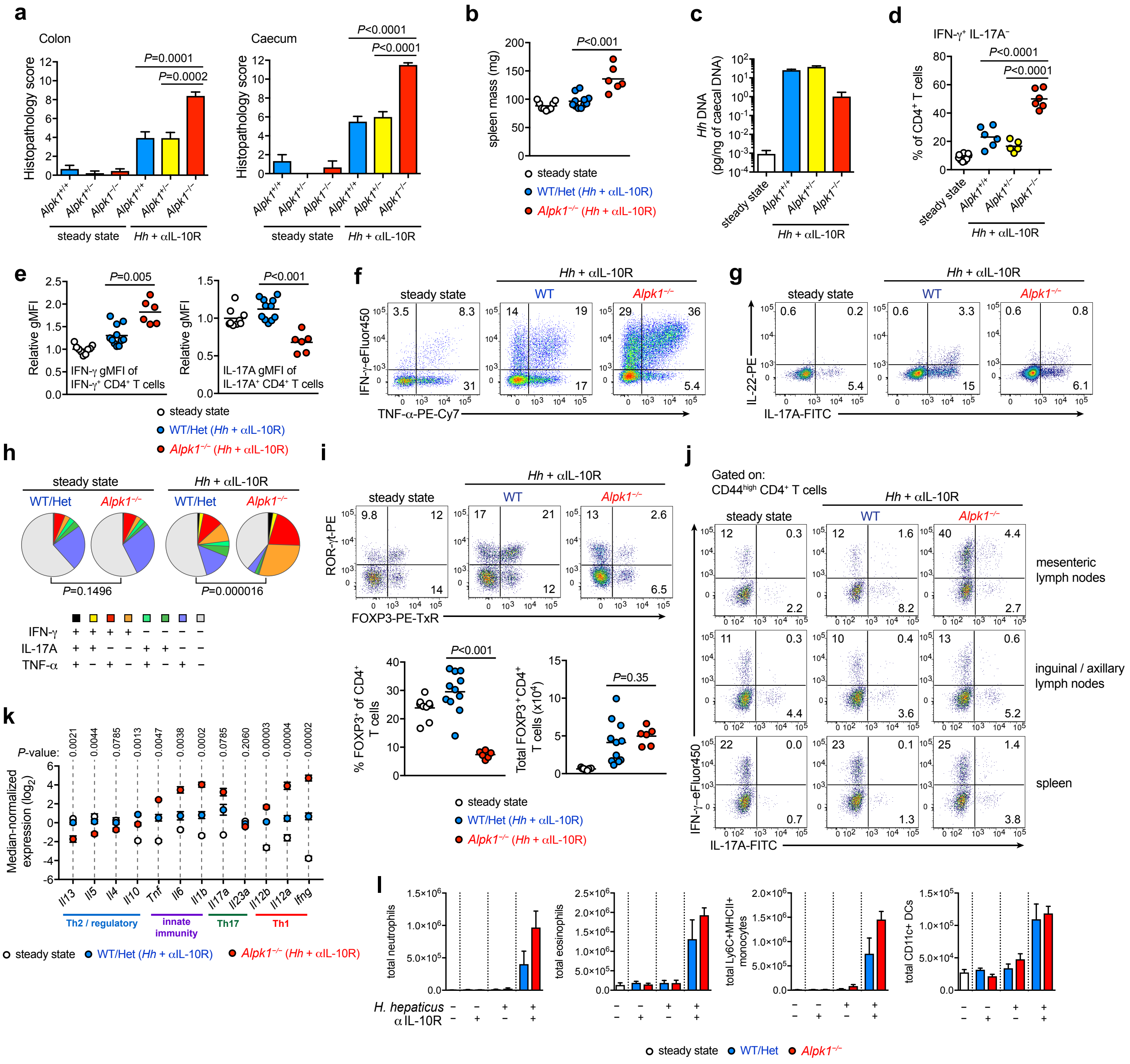


**Supplementary Figure 4. Regulation of colitis by Alpk1 in the *Hh* + αIL-10R model (associated with Fig. 3).**

(a-l) Molecular, cellular, and histological analysis of mice 12 days after induction of *Hh* + αIL-10R colitis. n=9 steady state mice (n=3 of each genotype), n=6 *Alpk1*$^{+/+}$, n=5 *Alpk1*$^{+/-}$ (combined WT/HET, n=11), and n=6 *Alpk1*$^{-/-}$ colitic mice. Data summarize at least two independent experiments.

(a) Colon and caecum histopathology scores after 12 days of colitis (demonstrating that *Alpk1*$^{+/-}$ and *Alpk1*$^{+/+}$ mice are phenotypically identical). Significance determined using one-way ANOVA.

(b) Spleen mass of steady state or *Hh*-infected mice. Significance determined by Mann-Whitney tests for *Hh*-treated groups only.

(c) *Hh* colonization of the caecum, approximated by quantifying *Hh* genomic DNA within DNA extracted from total caecal contents. *Hh* DNA was quantified by RT-qPCR analysis of the *cdtb* gene.

(d) Percentages of IFN-γ$^{+}$ IL-17A$^{-}$ cells among colon CD4$^{+}$ T cells. There were no significant differences between *Alpk1*$^{+/-}$ and *Alpk1*$^{+/+}$ mice.

**Supplementary Figure 4 (continuing).**

(e) Relative gMFI of IFN-γ and IL-17A in $CD4^+$ IFN-$\gamma^+$ and $CD4^+$ IL-$17A^+$ T cells, respectively. Data analyzed by Mann-Whitney test for *Hh*-treated groups only.

(f-g) Representative flow cytometry plots of IFN-γ, TNF, IL-17A, and IL-22 expression by colonic $CD4^+$ T cells after stimulation with PMA/ionomycin and brefeldin-A.

(h) SPICE analysis of IFN-γ, IL-17A, and TNF co-expression in colonic $CD4^+$ T cells. Pies represent the frequency of cells expressing the indicated combination of cytokines.

(i) Representative staining of the FOXP3 and RORγt transcription factors in colonic $CD4^+$ T cells. The percentage of $FOXP3^+$ Tregs among $CD4^+$ T cells and their total numbers are provided. Significance determined by Mann-Whitney test for *Hh*-treated groups only.

(j) IFN-γ and IL-17A expression among memory/activated ($CD44^{high}$) $CD4^+$ T cells from the mesenteric lymph nodes, peripheral lymph nodes (inguinal + axillary), and spleen.

(k) Whole colon gene expression analysis using RT-qPCR. Data points represent means +/− SEM. P-values represent comparisons between WT/Het and *Alpk1*$^{-/-}$ colitic mice, calculated using t-tests with corrections for multiple testing using the Holm-Sidak method.

(l) Plots displaying numbers of neutrophils ($CD11b^{high}$ $Gr1^{high}$), inflammatory monocytes ($Ly6C^{high}$ $MHCII^+$), eosinophils ($CD11b^+$ $SiglecF^+$) and $CD11b^-$dendritic cells ($CD11b^-$ $CD11c^+$ $MHCII^+$) in colon LPL of *Alpk1*$^{+/-}$ and *Alpk1*$^{-/-}$ mice at steady state (n=6), or after 2 weeks of treatment with IL-10R neutralizing antibody (*Alpk1*$^{+/-}$ n=6; *Alpk1*$^{-/-}$ n=4), infection with *Hh* (*Alpk1*$^{+/-}$ n=7; *Alpk1*$^{-/-}$ n=6), or treatment with anti-IL-10R antibody and infection with *Hh* (*Alpk1*$^{+/-}$ n=4; *Alpk1*$^{-/-}$ n=4). Data represent 2 independent experiments. Bar charts indicate means +/– SEM.

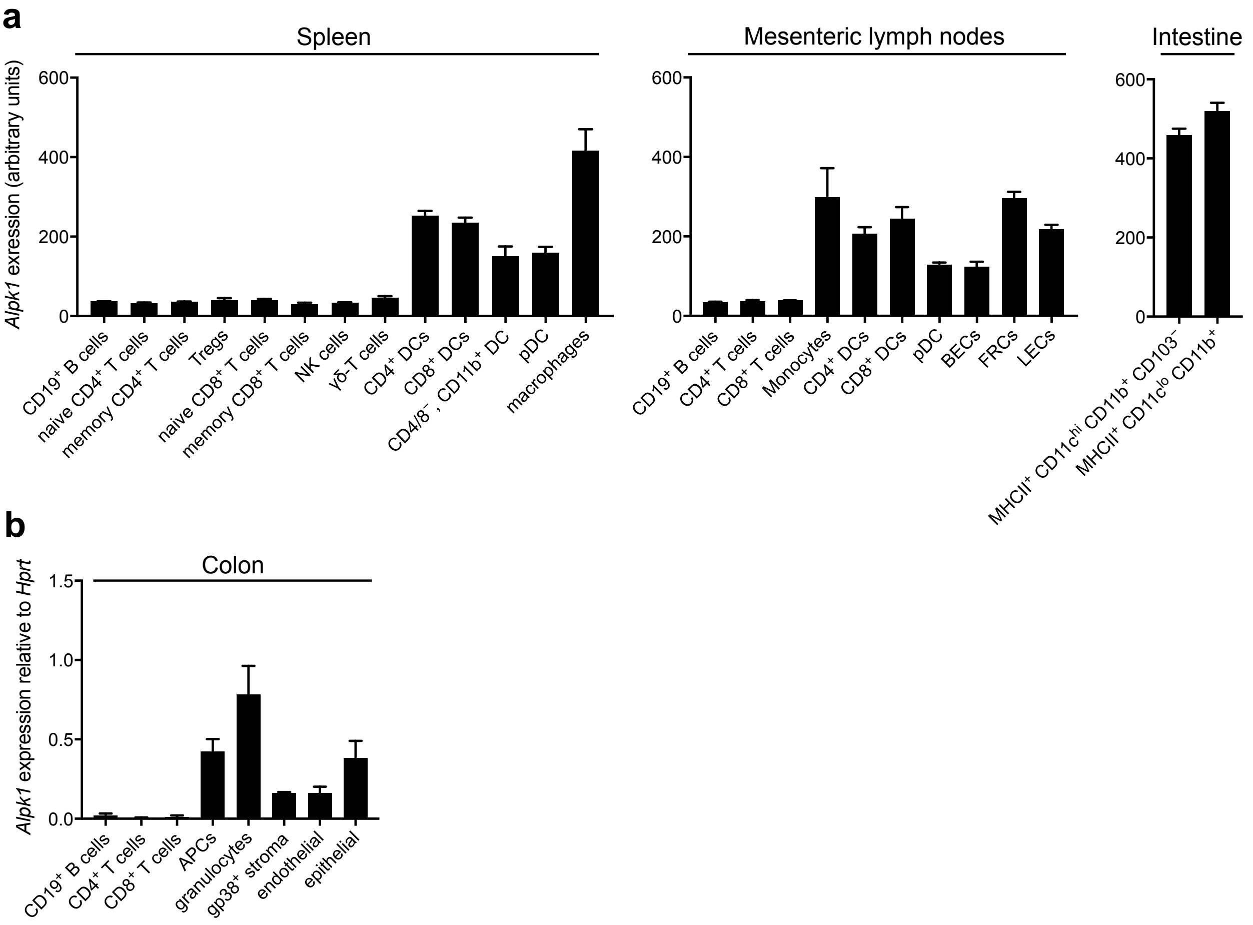


**Supplementary Figure 5. Alpk1 expression in different mouse cell types and organs.**

(a) Analysis of gene expression array data downloaded from the ImmGen database. Bars represent mean (+/− SEM) Alpk1 expression in arbitrary units from the indicated FACS-purified cell populations (n=2–12 per cell type). BEC, blood endothelial cell; DC, dendritic cell; FRC, fibroblastic reticular cell; LEC, lymphatic endothelial cell; pDC, plasmacytoid dendritic cell.

(b) *Alpk1* expression assessed by RT-qPCR from FACS-purified steady state C57BL/6 mouse colon cell populations sorted in-house (n=4). Granulocytes include both eosinophils and neutrophils. APCs (antigen presenting cells) include both macrophages and dendritic cells.

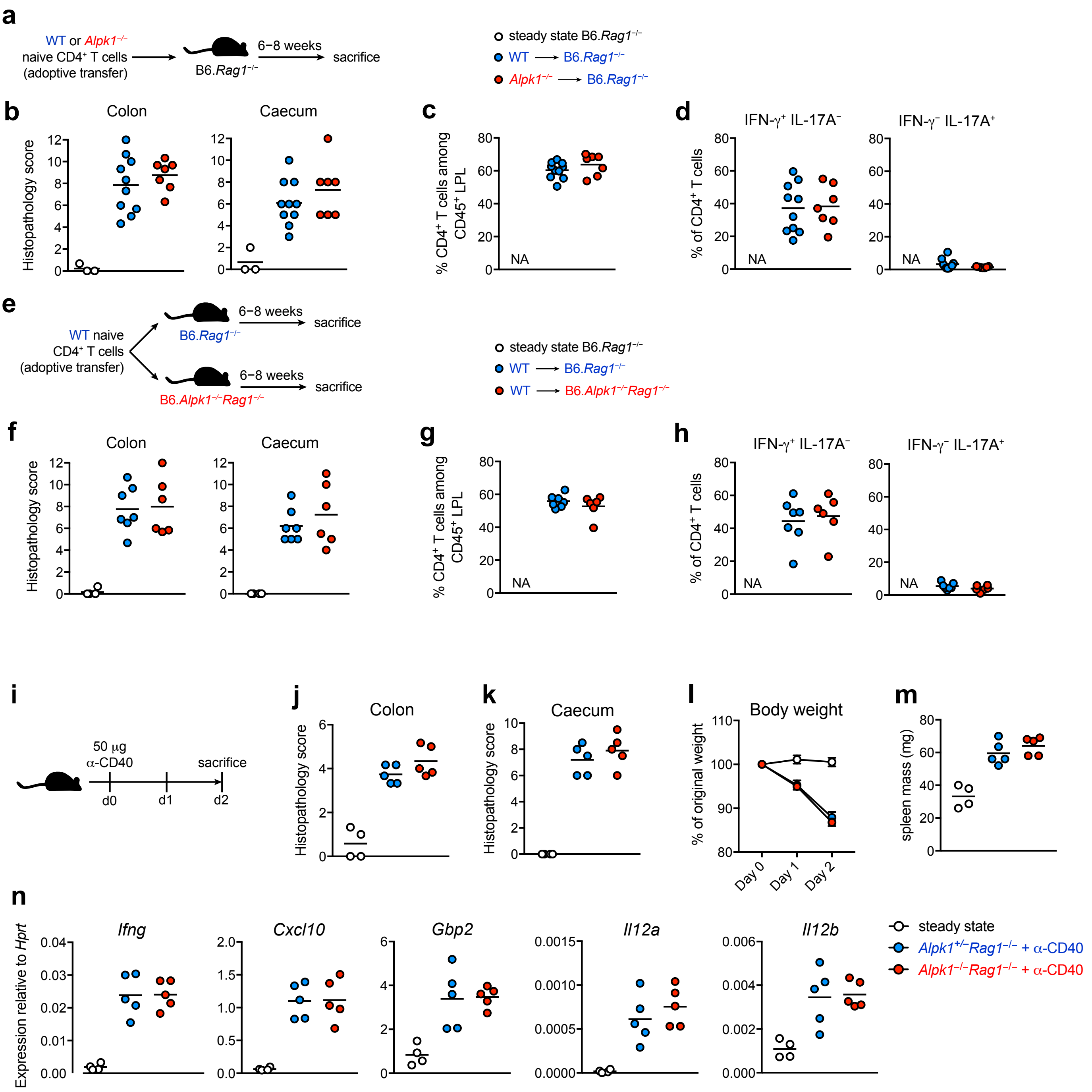


**Supplementary Figure 6. Alpk1 does not affect anti-CD40 mediated colitis or colitis driven by adoptive transfer of naïve T cells into lymphocyte-deficient hosts.**

(a) Experimental scheme for testing the colitogenic potential of wild type or *Alpk1*$^{-/-}$ naïve T cells transferred to *B6.Rag1*$^{-/-}$ mice (applies to panels b–d). One experiment was performed using *B6.Rag1*$^{-/-}$ mice (steady state, n=3; recipients of *Alpk1*$^{+/+}$ T cells, n=10; recipients of *Alpk1*$^{-/-}$ T cells, n=7).

(b) Colon and caecal histopathology scores.

(c) Frequency of CD4$^{+}$ T cells among total CD45$^{+}$ colon LPL.

(d) Frequencies of IFN-γ$^{+}$ IL-17A$^{-}$ and IFN-γ$^{-}$ IL-17A$^{+}$ cells among colonic CD4$^{+}$ T cells.

(e) Experimental scheme for testing the colitic potential of wild type naïve CD4$^{+}$ T cells transferred to *Alpk1*$^{+/+}$*Rag1*$^{-/-}$ or *Alpk1*$^{-/-}$*Rag1*$^{-/-}$ mice (applies to panels f–h). One experiment was performed using steady state B6.*Rag1*$^{-/-}$ (n=3), B6.*Rag1*$^{-/-}$*Alpk1*$^{-/-}$ (WT T cell-transfer, n=6) and B6.*Rag1*$^{-/-}$*Alpk1*$^{+/+}$ mice (WT T cell-transfer, n=7).

(f) Colon and caecal histopathology scores.

(g) Frequency of CD4$^{+}$ T cells among total CD45$^{+}$ colon LPL.

(h) Frequencies of IFN-γ$^{+}$ IL-17A$^{-}$ and IFN-γ$^{-}$ IL-17A$^{+}$ cells among colonic CD4$^{+}$ T cells.

**Supplementary Figure 6 (continuing).**

(i) Experimental scheme depicting treatment of B6.*Rag1*$^{-/-}$ mice with an agonistic anti-CD40 antibody to induce acute innate colitis. Mice were sacrificed after 2 days (applies to panels j-n). One experiment was performed using steady state B6.*Rag1*$^{-/-}$ (n=4), and anti-CD40-treated B6.*Rag1*$^{-/-}$*Alpk1*$^{+/-}$ (n=5) and B6.*Rag1*$^{-/-}$*Alpk1*$^{-/-}$ mice (n=5).

(j-m) Colon and caecum histopathology, body weight, and spleen weight.

(n) Whole colon gene expression analysis by RT-qPCR.

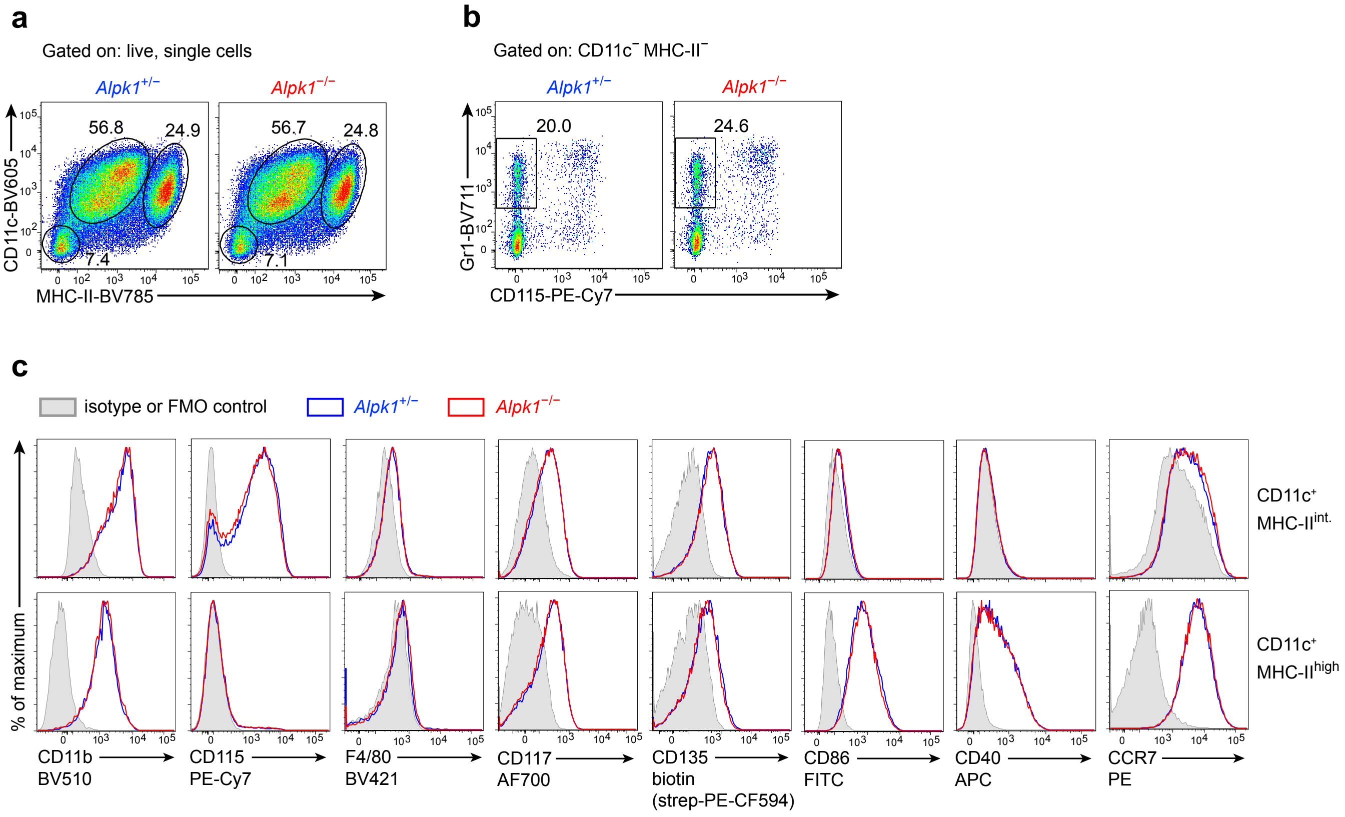


**Supplementary Figure 7. Comparable lineage marker expression in wild type and Alpk1 KO *in vitro* differentiated BMDMs.**

(a-c) Representative flow cytometry plots depicting expression of lineage surface markers in wild type and *Alpk1*$^{-/-}$ BMDMs. Data summarise two independent experiments.

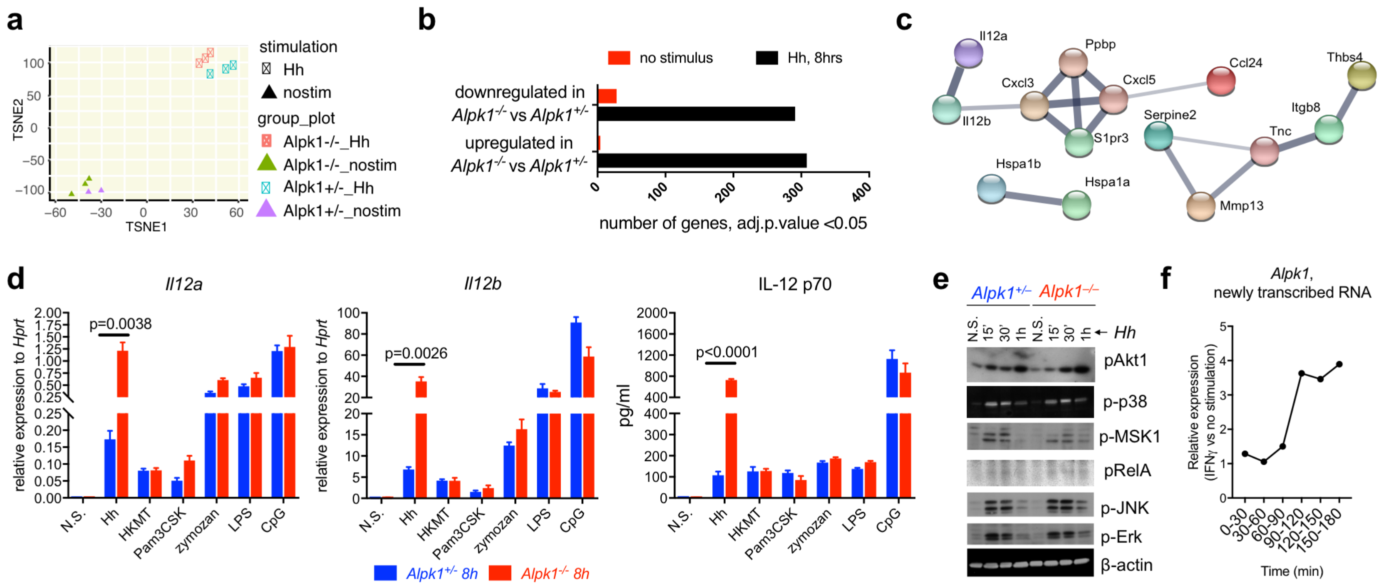


**Supplementary Figure 8. Alpk1 control of IL-12 expression occurs at the transcriptional level and specifically in *Hh* but not PAMP-stimulated mouse BMDMs**

(a) t-Stochastic Neighbour Embedding (tSNE) analysis of RNA-Seq samples (based on the 10345 genes that show significant variation between the experimental conditions). Data shown are from pooled bone marrows (n=3 per genotype) from a single experiment (a-c).

(b) Comparison of numbers of differentially expressed genes between the genotypes in unstimulated vs *Hh*-treated cells.

(c) STRING[1] clustering analysis of top 40 genes upregulated in *Hh*-treated *Alpk1*$^{-/-}$ cells compared to *Alpk1*$^{+/-}$ cells.

(d) IL-12 mRNA and protein levels in mouse *in vitro* differentiated BMDMs treated with microbial products. n=3 animals per genotype. Data summarise two independent experiments, analysed by unpaired t-test for *Hh*-treated group only (t=6.032 (*Il12a*), 6.705 (*Il12b*) and 24,35(IL-12-p70), df=4). HKMT, heat-killed *Mycobacterium tuberculosis*; CpG, Cytosine-phosphate-Guanine, a TLR9 agonist; LPS, lipopolysaccharide.

(e) Western blot analysis of protein phosphorylation induced in *Hh*-treated mouse *in vitro* differentiated *Hh*-treated BMDMs. Representative images from two independent experiments.

(f) The kinetics of *Alpk1* mRNA induction by IFNγ in mouse BMDMs, from the publicly available dataset GSE63290[2].


1 Snel, B., Lehmann, G., Bork, P. & Huynen, M. A. STRING: a web-server to retrieve and display the repeatedly occurring neighbourhood of a gene. *Nucleic Acids Res* **28**, 3442-3444 (2000).

2 Robertson, K. A. *et al.* An Interferon Regulated MicroRNA Provides Broad Cell-Intrinsic Antiviral Immunity through Multihit Host-Directed Targeting of the Sterol Pathway. *PLoS Biol* **14**, e1002364, doi:10.1371/journal.pbio.1002364 (2016).

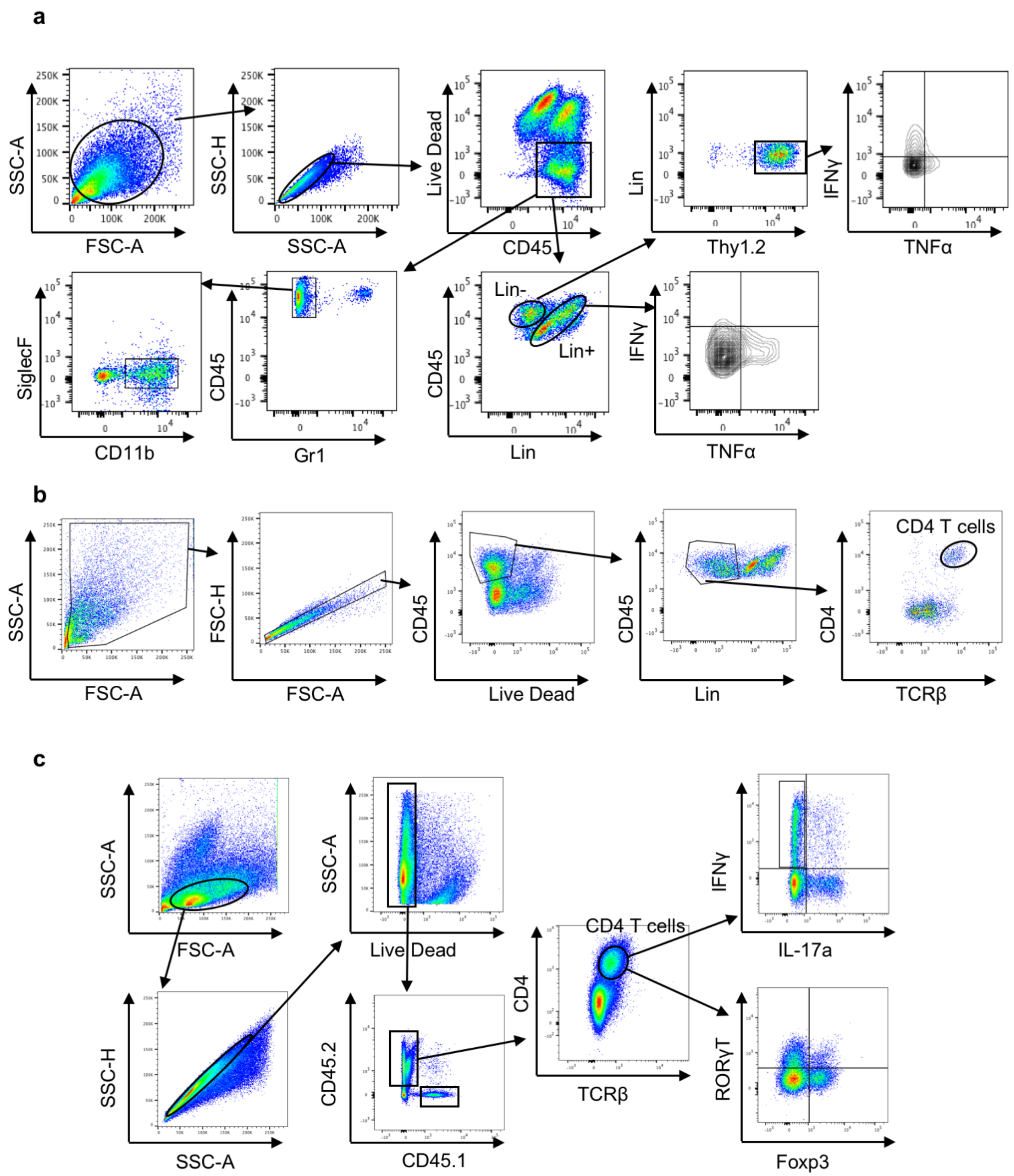


**Supplementary Figure 9. FACS gating strategies used for immune phenotyping.**

(a) Gating strategy to define Lin$^{-}$Thy1$^{+}$ (ILCs) and Lin$^{+}$ (myeloid) cells presented on Fig. 2e and CD45$^{+}$Gr1$^{low}$SiglecF$^{-}$CD11b$^{+}$ cells in innate colitis experiments presented on Suppl. Fig.3a,e. Lin$^{+}$ is defined as B220$^{+}$CD11c$^{+}$CD11b$^{+}$ population.

(b) Gating strategy to define CD4 T cell subset in Hh infection experiments presented on Figure 3f,j and Suppl. Fig. 4f,g,i,j.

(c) Gating strategy to define CD4 T cell subset in bone marrow chimera experiments presented on Fig. 4b-f.
