# Supplementary material for: Alpha kinase 1 controls intestinal inflammation by suppressing the IL-12/Th1 axis
Source: Nat Commun. 2018 Sep 18;9:3797. doi: 10.1038/s41467-018-06085-5 (PMC6143560; doi:10.1038/s41467-018-06085-5)
Supplement: Supplementary file 3 — Description of Additional Supplementary Files [file 41467_2018_6085_MOESM3_ESM.pdf]

Description of Additional Supplementary Files

Supplementary Data 1
Description: Transcriptome profiling of gene expression in the colon tissues of steady state and Helicobacter hepaticus-infected 129SvEvS6.Rag2−/− mice and the congenic strain 129.C3B. Rag2−/− R17.

Supplementary Data 2
Description: Whole transcriptome profiling of the steady state and Hh-treated (8 hrs) Alpk1+/- and Alpk1-/- BMDMs using RNA-Sequencing approach.
